# Glycosylinositol phosphorylceramides from *Rosa* cell cultures are boron-bridged in the plasma membrane and form complexes with rhamnogalacturonan II

**DOI:** 10.1111/tpj.12547

**Published:** 2014-06-17

**Authors:** Aline Voxeur, Stephen C Fry

**Affiliations:** The Edinburgh Cell Wall Group, Institute of Molecular Plant Sciences, The University of EdinburghEdinburgh, EH9 3JH, UK

**Keywords:** boron, cross-linking, glycosylinositol phosphorylceramides, glycosylinositol phosphoceramides, lipid rafts, rhamnogalacturonan II, cell wall, plasma membrane, *Rosa* sp

## Abstract

Boron (B) is essential for plant cell-wall structure and membrane functions. Compared with its role in cross-linking the pectic domain rhamnogalacturonan II (RG-II), little information is known about the biological role of B in membranes. Here, we investigated the involvement of glycosylinositol phosphorylceramides (GIPCs), major components of lipid rafts, in the membrane requirement for B. Using thin-layer chromatography and mass spectrometry, we first characterized GIPCs from *Rosa* cell culture. The major GIPC has one hexose residue, one hexuronic acid residue, inositol phosphate, and a ceramide moiety with a C_18_ trihydroxylated mono-unsaturated long-chain base and a C_24_ monohydroxylated saturated fatty acid. Disrupting B bridging (by B starvation *in vivo* or by treatment with cold dilute HCl or with excess borate *in vitro*) enhanced the GIPCs’ extractability. As RG-II is the main B-binding site in plants, we investigated whether it could form a B-centred complex with GIPCs. Using high-voltage paper electrophoresis, we showed that addition of GIPCs decreased the electrophoretic mobility of radiolabelled RG-II, suggesting formation of a GIPC–B–RG-II complex. Last, using polyacrylamide gel electrophoresis, we showed that added GIPCs facilitate RG-II dimerization *in vitro*. We conclude that B plays a structural role in the plasma membrane. The disruption of membrane components by high borate may account for the phytotoxicity of excess B. Moreover, the *in-vitro* formation of a GIPC–B–RG-II complex gives the first molecular explanation of the wall–membrane attachment sites observed *in vivo*. Finally, our results suggest a role for GIPCs in the RG-II dimerization process.

## Introduction

Normal plant growth and development require the element boron (B) (Warington, 1923; Blevins and Lukaszewski, 1998; Goldbach and Wimmer, 2007), although our current understanding of the biochemical basis of this B dependency lacks important detail and needs to be explored from several novel perspectives. B deprivation causes many anatomical, physiological and biochemical changes and, because of the rapidity and the wide variety of symptoms that follow B deficiency, determining the primary function of B in plants is one of the greatest challenges in plant nutrition. Why excess B is highly toxic to plants is also a mystery (Aquea *et al*., 2012). B deficiency leads to the development of brittleness of tissues (Loomis and Durst, 1992), abnormal cell-wall thickness, decreased root cell-wall elasticity (Findeklee and Goldbach, 1996) and pollen-tube growth deceleration within 2 min of B withdrawal. Collenchyma, which is particularly rich in pectin, is strongly compromised by B deficiency; conversely, the Poales, which are poor in pectin, have a low B requirement (Hu *et al*., 1996). Thus, a special significance of pectins in the function of B is widely accepted.

Pectins are complex acidic polysaccharides of the primary cell wall that contain at least three major domains: homogalacturonan, rhamnogalacturonan I (RG-I) and RG-II. Of these, RG-II is the most complex, being composed of a short α-(1→4)-linked homogalacturonan backbone substituted with five structurally different side chains (the oligosaccharides A and B, disaccharides C and D, and arabinose) and is described as the main, and maybe exclusive, cell-wall binding site for B (Matoh *et al*., 1996). Much of the RG-II in the cell wall is usually present as a dimer cross-linked by a borate diester *via* the *cis*-diol groups of two apiose (Api) residues of side-chain A (O’Neill *et al*., 1996). The structure of RG-II is highly conserved between species (Matsunaga *et al*., 2004; O’Neill *et al*., 2004), with relatively minor variation (Pabst *et al*., 2013), and it has been shown that modification of its structure affects its ability to dimerize, correlating with severe growth phenotypes and pollen-tube growth defects (O’Neill *et al*., 2001; Ahn *et al*., 2006; Delmas *et al*., 2008; Voxeur *et al*., 2011).

Much evidence suggests that B also plays a role in the structure and function of the plant plasma membrane (Loomis and Durst, 1992; Blevins and Lukaszewski, 1998). Within minutes, B deprivation inhibits P_i_ and Rb^+^ uptake (Pollard *et al*., 1977; Goldbach, 1985) and ferricyanide-induced H^+^ release (Goldbach *et al*., 1990), suggesting that B does not act only by maintaining wall integrity. Withholding B also causes visible abnormalities at the wall–membrane interface and is likely to be involved in the organisation of transvacuolar cytoplasmic strands and/or to participate in wall–membrane attachment (Hirsch and Torrey, 1980; Bassil *et al*., 2004). It has also been proposed that B could be involved in the structure of lipid rafts by forming cross-links with *cis-*diol groups present in raft components (Wimmer *et al*., 2009) such as the mannose residues in GPI-anchored proteins and the sugar residues of glycolipids, thereby dictating the membrane’s physical state (Brown *et al*., 2002). Together, these observations suggest that a plant-specific, B-dependent membrane component may exist, although it is yet to be isolated.

Glycosylinositol phosphorylceramides (GIPCs) are the major sphingolipids in the plant plasma membrane (Markham *et al*., 2006, 2013), especially in lipid rafts (Borner *et al*., 2005). They are encountered only in plants and fungi, including yeasts (Sperling *et al*., 2005). One characteristic feature of GIPCs is the high degree of hydroxylation of the fatty acid and the long-chain base that compose their ceramide moiety (Lester and Dickson, 1993; Lynch and Dunn, 2004). The fatty acids of GIPCs are very long-chain moieties (C_22_ to C_26_) that contain a C-2 hydroxyl group (Lynch and Dunn, 2004). The long-chain base typically contains 18 carbon atoms and three hydroxyl groups: the C-1 and the C-3 hydroxyl groups arise from serine and palmitoyl-CoA precursors, respectively, whereas the C-4 hydroxyl group is subsequently added by a sphingoid base hydroxylase. Unlike those of fungi, including yeasts, plant GIPCs usually contain an acidic sugar (α-GlcA) linked to the inositol–phosphate–ceramide core structure; additional sugar units may also be attached to the GlcA. (Kaul and Lester, 1978; Buré *et al*., 2013).

In the present study, we investigated the role of B in the plasma membrane structure and tried to provide a better understanding of the role of B in wall–membrane attachment. First, using a thin-layer chromatography (TLC) and mass spectrometry (MS) approach, we characterized GIPCs from rose cell cultures. We discovered that B influences the extractability of GIPCs and that disruption of borate ester linkages led to the solubilisation of a detergent-insoluble fraction that includes lipid rafts. Finally, we showed that GIPCs are able to bind RG-II, possibly via a B bridge, and that they can favour the B-dependent dimerization of RG-II.

## Results

### GIPC characterization of rose cells

**Figure 1:**
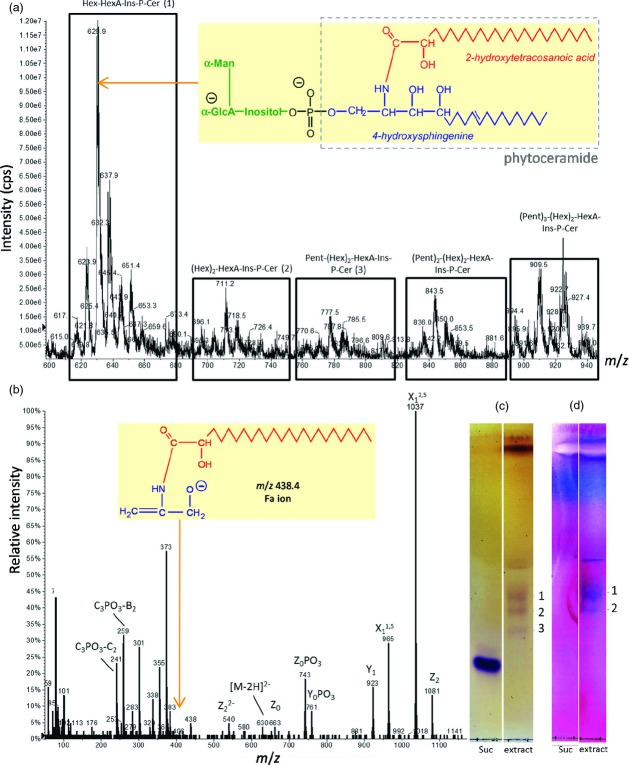
Mass spectrometric and thin-layer chromatographic analysis of *Rosa* glycosylinositol phosphorylceramides (GIPCs). (a) ESI-MS analysis of GIPC extract from *Rosa* cell culture. The spectrum was acquired in the negative ion mode. Abbreviations: Hex, hexose residue (probably α-mannose); HexA, hexuronic acid residue (probably α-glucuronic acid); Pent, pentose residue; Ins, *myo*-inositol; P, phosphate; Cer, phytoceramide. Inset: proposed structure of the predominant GIPC species; phytoceramide moiety in grey box. (b) ESI-MS/MS (collision-induced dissociation spectrum) analysis of the predominant Hex-HexA-Ins-P-Cer peak seen in (a) as the [M-2H]^2−^ ion at *m*/*z* 630. Nitrogen was used as collision gas in a Q-TRAP instrument, with the collision energy set to −40 eV. The standard nomenclature for glycolipid fragmentation has been applied (Costello and Vath, 1990; Levery *et al*., 2001). Inset: proposed identity of the ion at *m*/*z *=* *438.4, indicating an h24:0 ceramide moiety. (c, d) Thin-layer chromatography (TLC) of GIPC extract. Lipids were chromatographed in CHCl_3_/CH_3_OH/4 m NH_4_OH (9:7:2, by vol.) with 0.2 m ammonium acetate (Kaul and Lester, 1978) and located by orcinol reagent (c) or periodic acid–Schiff staining (d). Lipid bands are labelled: 1, Hex-HexA-Ins-P-Cer; 2, (Hex)_2_-HexA-Ins-P-Cer; 3, Pent-(Hex)_2_-HexA-Ins-P-Cer.

In order to characterize GIPCs from a rose cell culture, we extracted and analysed them according to a protocol described previously (Buré *et al*., 2011). Mass spectrometric analyses of GIPCs revealed five clusters of compounds (Figure1a). The most intense peak was found in the first cluster at *m*/*z* 629.9 ([M-2H]^2−^ ion) corresponding to a GIPC with one hexuronic acid residue, one hexose residue and a t18:1 h24:0 ceramide moiety (where t18:1 indicates a trihydroxylated long-chain base with 18 C atoms and one C=C bond, and h24:0 indicates a monohydroxylated fatty acid with 24 C atoms and no C=C bonds) and containing one ^13^C atom (out of the 60). The other peaks of this cluster were mainly attributed to mono-hexosylated GIPCs composed of long-chain base t18:0 and t18:1 and fatty acid chains h22:0 to h28:0. Ions of doubly charged species corresponding to the other clusters were assigned to dihexosylated GIPC (*m*/*z *=* *711.2), and the same containing up to three pentoses and thus having mainly a t18:1 h24:0 ceramide moiety (*m*/*z = *777.5, 843.5, 909.5). These structures were further confirmed by MS fragmentation analysis (Figure1b). The hexose and hexuronic acid present in the major GIPC were not identified; however, they are most likely to be α-d-mannose and α-d-glucuronic acid respectively because the same *Rosa* culture releases into its culture medium a GIPC-derived fragment which has been characterized as α-d-mannopyranosyl-(1→4)-α-d-glucuronopyranosyl-(1→2)-*myo*-inositol (Smith and Fry, 1999; Smith *et al*., 1999).

The separation of the lipid extract by TLC followed by orcinol staining to visualise the glycolipids revealed three low-mobility bands corresponding to GIPCs (Figure1c). Periodic acid–Schiff staining allowed the detection of the two most intense bands (bands 1 and 2; Figure1d) and of authentic commercial phytoceramide (not shown) but did not stain sucrose suggesting that the staining was specific to the *cis-*diol of the lipid moiety. According to the intensity of the bands obtained with the different stains and their relative mobility, the fastest-migrating band(band 1) was assigned to mono-hexosylated GIPCs, band 2 to dihexosylated GIPC and band 3 to GIPC containing one pentose. The average mono-hexosylated GIPC:dihexosylated GIPC ratio obtained from image analysis of TLCs stained by the periodic acid–Schiff method was around 4:1.

### Impact of boron on GIPC extraction

**Figure 2:**
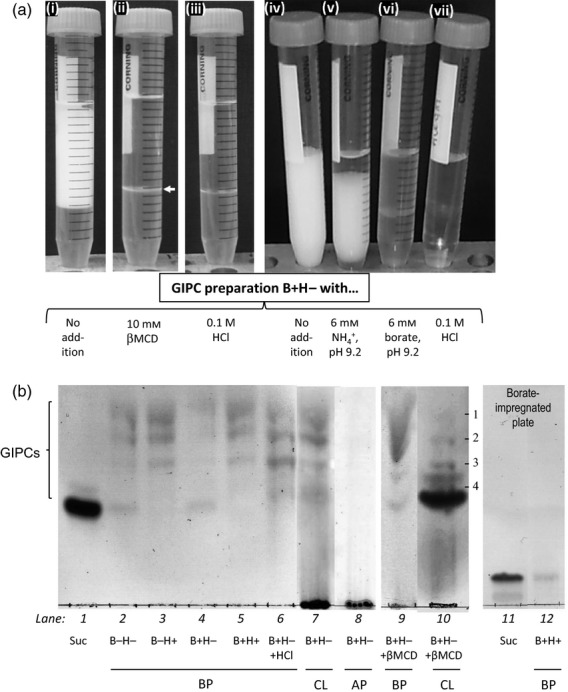
Influence of boron on glycosylinositol phosphorylceramide (GIPC) extraction. (a) A cloudy layer was observed during butanol/water phase-partitioning of a GIPC-enriched lipid sample extracted with neutral ethanol from *Rosa* cell cultures that had been grown in the usual B concentration (i, iv). This cloudy layer disappeared in the presence of 0.1 m HCl (iii, vii), 10 mm βMCD (ii), or 6 mm borate buffer, pH 9.2 (vi). The horizontal arrow indicates the slight cloudy layer left in the presence of βMCD (butanol above). In contrast, 6 mm ammonium buffer, pH 9.2 (v), only led to a partial disappearance. (b) TLC of the different phases after butanol/water phase-partitioning of a GIPC-rich lipid extract from *Rosa* cell cultures grown in media with (B+) or without boron (B−). The lipids had been extracted in 70% ethanol that contained 0.1 m HCl (H+) or lacking acid (H−). BP, butanol phase; CL, cloudy layer; AP, aqueous phase; Suc, sucrose (marker). In lanes 9 and 10, 10 mm βMCD was present during the partitioning step. Lipids labelled on lane 10: bands 1–3, as in Figure1; band 4, (Pent)_2_-(Hex)_2_-HexA-Ins-P-Cer.

To explore the putative existence of borate-bridged GIPC *in vivo,* we took advantage of aqueous solubility of GIPCs (Markham *et al*., 2006) and investigated the effect of boric acid (H_3_BO_3_) and HCl treatment on GIPC extractability. We extracted GIPCs from rose cell cultures grown with (B+) or without (B−) the routine concentration (3.3 μm) of H_3_BO_3_. Also, as cold 0.1 m HCl is able to hydrolyse the borate diester linkage in RG-II, we used acidic aqueous ethanol (H+) or non-acidic aqueous ethanol (H−). When B+ cultures were investigated, the use of H− as extractant resulted in the appearance of a prominent cloudy layer during the subsequent butan-1-ol/water phase-partitioning, interpreted as B-bridged lipid-rich material (Figure2a,d). This layer was less abundant in B−H−than in B+H−, and nearly absent in B−H+ and B+H+ (data not shown). Adding 0.1 m HCl to the B+H−sample during the butanol/water phase-partition step made this cloudy layer disappear (Figure2a-iii,vii). In the B−H+ and B+H+ samples (lacking a cloudy layer, as mentioned) no cloudiness subsequently formed after removal of the acidity by neutral acetone washes, suggesting that the disappearance of the cloudy layer was not simply pH dependent. As a consequence, a chemical modification such as the disruption of borate bridging must be responsible for the non-formation or the disappearance of the cloudy layer. Likewise, excess borate buffer, which could potentially disrupt B bridges (figure 7 of Bassil *et al*., 2004), solubilised all the cloudy layer, whereas ammonium buffer at the same concentration and pH did not (Figure2a-v,vi). Finally, 10 mm methyl β-cyclodextrin (βMCD), a cholesterol- and phytosterol-complexing agent that is capable of disrupting detergent-insoluble glycolipid-enriched complexes (potentially lipid rafts; Roche *et al*., 2008), solubilised most of, but not all, the cloudy layer (Figure2a-ii). The cloudy layer left was solubilised by addition of 0.1 m HCl.

As judged by TLC, more GIPC was present in the butanol phase (BP) obtained from B-deficient cell cultures with non-acidified ethanol (B−H−; Figure2b, lane 2) than in that obtained from control cell cultures (B+H−; Figure2b, lane 4). Using acidified ethanol clearly increased the GIPC amount present in the BP from the B+ extract (B+H+; Figure2b, lane 5) but not in that from B– preparation (B−H+, Figure2b, lane 3), suggesting that acid treatment interfered with the tethering of GIPC molecules within a lipid raft by disrupting potential borate ester linkages. Later addition of 0.1 m HCl to a previously neutral (B+H−) preparation during the phase-partition step also promoted the recovery of soluble GIPC in the BP (B+H−+HCl, Figure2b, lane 6). TLC of the compounds present in the cloudy layer of a never-acidified B+H−sample gave the same lipid profile as in the BP of a B+H+ sample (Figure2b, lane 7) but, in addition, high-molecular-weight (chromatographically immobile) carbohydrate-containing compounds were present. After acidification, these high molecular compounds were released into the aqueous phase (AP) (Figure2b, lane 8). Adding βMCD led to the partial recovery of GIPC in the BP (Figure2b, lane 9), and GIPCs associated with high-molecular-weight material were also found in the fraction of the cloudy layer (CL) that was non-solubilised by βMCD (Figure2b, lane 10). Interestingly, relatively more (Pent)_1 and 2_-(Hex)_2_-HexA-Ins-P-Cer (Figure2b, lane 10, bands 3 and 4) were found in the CL non-solubilised by βMCD compared with the native cloudy layer. The band below band 4 in lane 10 is unidentified, and probably arose from the βMCD.

**Figure 3:**
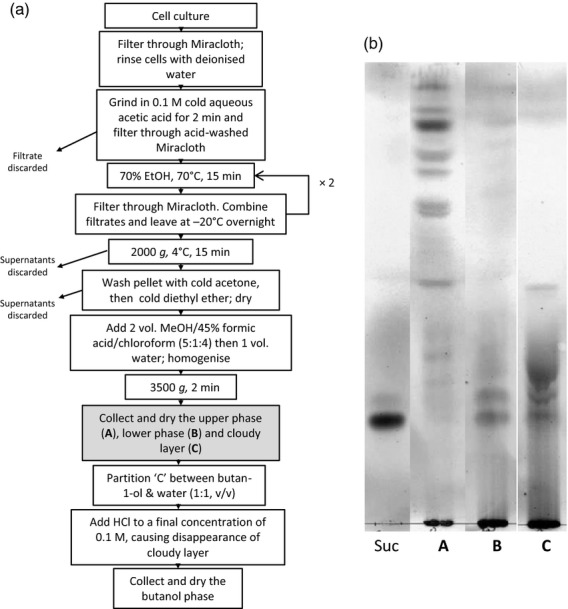
Purification of glycosylinositol phosphorylceramide (GIPC) from *Rosa* cell culture. (a) GIPC purification scheme adapted from Buré *et al*. (2011). (b) Thin-layer chromatography (TLC) of the phase A, B and C obtained during the step shaded grey in (a). Suc, sucrose marker.

Interestingly, the separation of the GIPC-enriched extracts on borate-impregnated silica gel plates showed a unique band of very low mobility instead of the four bands expected. The material in this band, assumed to be borate-cross-linked GIPCs, could not be eluted in butanol, suggesting that borate-bridged GIPCs were not soluble in butanol. They were, however, eluted in ethanol and when re-run on a borate-free TLC plate migrated with the same very low mobility, suggesting that the borate-bridged GIPC was stable.

### RG-II–GIPC binding assays

In order to test for possible RG-II–GIPC interactions, we tried to obtain GIPC as pure as possible by adapting the method of Buré *et al*. (2011). The main difference from the original protocol was the addition of a phase-partition step with methanol/chloroform/formic acid/water (Figure3a). As judged by TLC of the two phases obtained, the less polar lipids were found in the organic phase, the contaminating sugars in the AP and the GIPC at the interface (Figure3b). This interface material was then submitted to an acidic butanol/water phase partition, which removed polymers. The purified GIPC-containing BP was collected.

**Figure 4:**
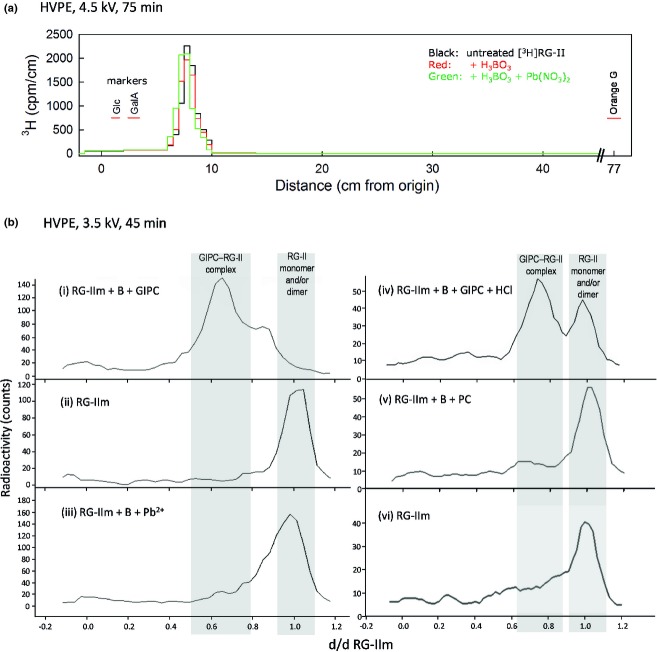
High-voltage paper electrophoresis of radiolabelled RG-II and the effect of boric acid, Pb^2+^ and glycosylinositol phosphorylceramides (GIPCs). (a) [^3^H]RG-II was loaded on to the paper after 9 h of pre-treatment of monomeric [^3^H]RG-II in 250 mm pyridine buffer, pH 4.7, with or without 1 mm H_3_BO_3_ or 1 mm H_3_BO_3_ plus 0.5 mm Pb(NO_3_)_2_. After electrophoresis, the paper was cut into strips, which were assayed for tritium by scintillation-counting. The positions of non-radioactive markers (glucose, galacturonic acid and Orange G) are also shown. (b) Samples were loaded on the paper after 4 h of pre-treatment of monomeric [^3^H]RG-II (RGIIm) in 50 mm ammonium acetate, pH 4.8, with or without various combinations of 1.2 mm boric acid (B), 0.5 mm Pb(NO_3_)_2_ (Pb^2+^), GIPCs (purified as described in Figure3), commercial phytoceramide (PC), and 0.1 m hydrochloric acid (HCl). After electrophoresis, papers were read on a LabLogic AR2000 radio-TLC Imaging Scanner. The distance migrated (d) is given relative to dRGIIm (the distance migrated by monomeric RG-II, that is to say approximately 6 cm). Grey bands indicate the positions of monomeric RG-II (right) and the GIPC–RG-II complex (left).

Using this extract, we investigated the ability of GIPC to bind RG-II by testing the effect of GIPC on the mobility of radiolabelled RG-II on paper electrophoresis. We incubated tracer quantities of [^3^H]RG-II with an excess of the purified GIPC under conditions suitable for RG-II dimerization (Chormova *et al*., 2013). On paper electrophoresis at pH 2.0, monomeric [^3^H]RG-II and the same preparation partially or fully dimerized by H_3_BO_3_, without or with 0.5 mm Pb^2+^ respectively, all migrated approximately 8 cm towards the anode (Figure4a). Thus dimeric RG-II had approximately 1.6× the charge of monomeric RG-II [estimated by application of Offord’s law, which states that mobility on paper electrophoresis is proportional to the *Q:M*_*r*_^2/3^ ratio, where *Q* is the molecule’s net charge and where the molecular weight to the power of 2/3 is an indication of the molecule’s surface area (Offord, 1966; Fry, 2011)]. Co-migration of monomeric and dimeric RG-II is confirmed in Figure4b-ii,iii).

Incubation of monomeric [^3^H]RG-II with GIPC caused a decrease in the electrophoretic mobility of the radioactive moiety. It gave a major peak with a shoulder, suggesting the existence of two species resulting from the interaction of RG-II with GIPC (Figure4b-i). Similar results were obtained when B was not deliberately added to the reaction mixture (data not shown), suggesting that enough B for RG-II–GIPC cross-linking was already present in the samples; but pre-treatment of the mixture with 0.1 m HCl for 1 h led to the partial re-formation of free [^3^H]RG-II (Figure4b-iv), compatible with the cleavage of borate bridges. To test the specificity of the GIPC, the same experiment was performed either with a *Rosa* lipid extract that did not contain GIPC (data not shown) or with authentic commercial phytoceramide (Figure4b-v). None of these lipids induced any change in the electrophoretic mobility of the [^3^H]RG-II, implying a special affinity of GIPC for RG-II.

### Impact of GIPC on RG-II dimerization

**Figure 5:**
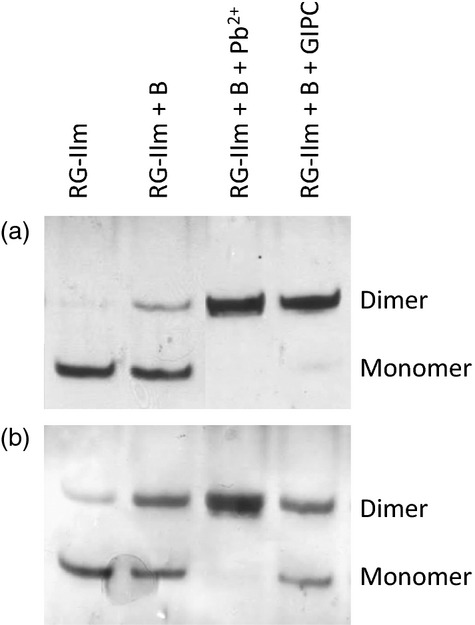
Glycosylinositol phosphorylceramide (GIPC)-induced dimerization of monomeric RG-II. After 4 h of pre-treatment of monomeric RG-II without or with 1.2 mm H_3_BO_3_ ± GIPC or with 1.2 mm H_3_BO_3_ and 0.5 mm Pb(NO_3_)_2_, samples were analysed by polyacrylamide gel electrophoresis and silver stained. Abbreviations as in Figure4. Parts (a) and (b) show the results of two independent experiments.

In order to test whether GIPC plays a role in RG-II dimerization, we incubated GIPC with purified RG-II plus H_3_BO_3_ under conditions devised for demonstrating Pb^2+^-induced dimerization. After 4 h, the reaction mixture was analysed by polyacrylamide gel electrophoresis (PAGE) (Chormova *et al*., 2013). Some experiments showed that GIPC was able to enhance dimer formation as effectively as Pb^2+^ *in vitro* (Figure5a). Other repeat experiments demonstrated a smaller effect of GIPC (Figure5b), although the dimerization rate was always higher with GIPC + H_3_BO_3_ than with H_3_BO_3_ alone.

## Discussion

### Characterization of GIPC from rose cell cultures

To characterize biochemically GIPCs from rose cell culture, we analysed the GIPC-enriched extract by MS. Five clusters of species were identified corresponding to GIPCs that contained one hexuronic acid residue (HexA; most likely α-d-glucuronic acid), one or two hexose residues (Hex; at least one of which is probably α-d-mannose), and from one to three pentose residues (Pent), agreeing with the polar head variability recently reported by Cacas *et al*. (2013). As relative abundance of different GIPC species could not be assessed on the basis of full-scan mass spectra (Buré *et al*., 2011), and as the relative abundance of GIPCs could not be accurately assessed on TLC by orcinol staining because of the variable number and composition of sugar units, we developed a TLC staining method based on periodic acid–Schiff staining. This method allowed the instantaneous staining of lipids containing a vicinal diol whereas no band appeared for the sucrose suggesting that, under the conditions used, this stain was specific for the *cis*-diol of the lipid moiety. The average mono-hexosylated GIPC/dihexosylated GIPC ratio estimated by this method was roughly 4:1, which is consistent with previous results in *Arabidopsis* (Mortimer *et al*., 2013).

### Structural role of boron in the plasma membrane

Boron deficiency has been shown to disrupt the membrane transport of P_i_ and Rb^+^ and the activity of membrane-localised proteins such as ATPase (Robertson and Loughman, 1974; Pollard *et al*., 1977; Smyth and Dugger, 1980; Goldbach, 1985). Interestingly, amongst proteins specifically detected in the detergent-insoluble (lipid-raft-associated) fractions of tobacco BY-2 cells, isoforms of H^+^-ATPase or subunits of vacuolar H^+^-ATPases and three isoforms of P_i_ transporter have been identified (Morel *et al*., 2006). We therefore suggest that an alteration of membrane structure and perhaps of lipid raft structure, caused by a B deficiency, could be responsible for the observed effects. As the role of B in membrane structure had never been directly demonstrated and as GIPCs have interesting *cis*-diol groups that are able to form borate ester bridges, we investigated the effect of boron on GIPC extractability.

We showed that GIPCs were more extractable from cells grown without B than from cells grown under normal conditions and that stripping of B with HCl improved GIPC recovery, suggesting that B influences the GIPC extractability. Moreover, the use of non-B-stripping extractants (neutral 70% ethanol) led to the appearance of a GIPC-containing CL during butanol/water phase-partitioning. This phenomenon, which was more pronounced in cells grown with B, is characteristic of micelle aggregation. The addition of βMCD [which has been reported to disrupt lipid rafts (Roche *et al*., 2008)] led to the almost total solubilisation of the CL, suggesting that this layer was mainly composed of lipid raft-containing micelles. Interestingly, the disruption of potential borate cross-links by excess borate or 0.1 m HCl also led to the total solubilisation of the CL. Together, these data indicate a potential structural role for B in the plasma membrane, possibly in lipid rafts. Furthermore, more (Pent)_*n*_-(Hex)_2_-HexA-Ins-P-Cer than Hex-HexA-Ins-P-Cer was found in the small quantity of the CL that was *not* solubilised by βMCD than in the native cloudy layer. This observation suggests that the major GIPCs that bind B *in vivo* are the highly glycosylated species.

As we showed that excess borate can solubilise GIPCs from the detergent-insoluble CL, we suggest a possible basis for the phytotoxicity of soils that have a high concentration of soluble B, which are prevalent in over-irrigated semi-arid areas (Al-Mustafa *et al*., 1993). Although a small amount of B appears to be required for the structural integrity of the membrane in plants, serving to cross-link GIPCs to other membrane components (possibly glycoproteins), excess B may interfere with such cross-linking. High B may independently bond to the two organic partners such that a single B can no longer cross-link them. We suggest that, at high B concentrations, B–GIPC and B–glycoprotein bonds form separately, precluding the formation of GIPC–B–glycoprotein bridges and thus interfering in the assembly of the membrane and perhaps of lipid rafts.

### GIPC–RG-II interactions

Previous work by Bassil *et al*. (2004) showed that B is involved in cell-wall–membrane attachment. Furthermore, fumonisin B1 (FB1), which specifically inhibits the incorporation of very-long-chain fatty acids into sphingolipids, caused a detachment of the plasma membrane (Markham *et al*., 2011), suggesting a role of GIPCs in the wall–membrane attachment. As RG-II is the only strong B-binding site in the cell wall, we investigated whether an RG-II–GIPC complex could be formed and potentially involved in the wall–membrane attachment. Using radioactive RG-II and high-voltage paper electrophoresis, we demonstrated that GIPCs, unlike other lipids tested, were able to form complexes with RG-II. HCl (0.1 m) disrupted the complex, as it does with RG-II–B–RG-II complexes, again supporting the existence of GIPC–B–RG-II complexes; however, it remains to be shown directly whether B was involved in the GIPC–RG-II complex. The RG-II–GIPC complex appears to be reasonably stable under acidic conditions, as it remained intact during about 1 h of electrophoresis at pH 2.0. The quantity of [^3^H]RG-II used in this study was very small, so the traces of soluble B present in ordinary laboratory buffers may have been sufficient for GIPC–RG-II bonding, explaining why exogenous H_3_BO_3_ did not modify the results obtained.

Analysis of wall-localised RG-II consistently shows the presence of 10–15% in the monomeric form, even in plants with an adequate boron supply (Matsunaga and Ishii, 2006). It is possible that this percentage represents RG-II molecules which, *in vivo,* had been present as a GIPC–RG-II complex but released in free form during the alkali treatment that is routinely applied before endopolygalacturonase digestion. Although that idea is speculative, some clues pointing to the existence of a GIPC–RG-II complex can be found in the literature. It has been discovered, by use of anti-RG-II antiserum in *Pisum sativum* symbiotic root nodules developed in presence of B, that RG-II interacted with a 175-kDa arabinogalactan-protein–extensin (AGPE) hybrid glycoprotein (Reguera *et al*., 2010). Some AGPs, owing to their glycosylphosphatidylinositol (GPI) lipid anchor, are associated with detergent-resistant membrane (Seifert and Roberts, 2007). Thus, it is likely that some lipids such as GIPCs, tightly associated with the GPI anchor, could be present in the 175-kDa nodule complex containing RG-II antigen. Moreover, immunocytochemical studies using RG-II-specific antibodies demonstrated a denser label proximal to the plasma membrane (Matoh *et al*., 1998), suggesting that RG-II could be bound to the membrane. These previous results strengthen the possible presence of a GIPC–B–RG-II complex *in vivo*.

The dimerization of RG-II through B bridges occurs only very slowly when RG-II and H_3_BO_3_ are mixed *in vitro,* but the process can be greatly expedited by addition of certain metal ions, especially Pb^2+^ or Sr^2+^ (O’Neill *et al*., 1996; Ishii *et al*., 1999). Boron-mediated cross-linking of newly synthesised RG-II occurs very rapidly *in vivo* (Chormova *et al*., 2013). The factors that promote the cross-linking *in vivo* are unknown, but it is clear that they do not normally include Pb^2+^ or Sr^2+^, which are not essential elements. What biological agent ‘mimics’ Pb^2+^ *in vivo?* One attractive possibility, suggested by the present work, is that a GIPC serves as a B ligand (**L**) promoting boryl-transfer to RG-II, in reactions of the type:
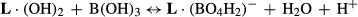
Reaction 1
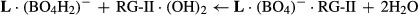
Reaction 2
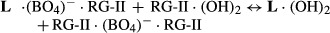
Reaction 3

In reaction 1, boric acid binds to the ligand (GIPC); in reaction 2, the GIPC forms a B-centred complex with RG-II; then in reaction 3, the GIPC is displaced by an incoming second RG-II molecule, resulting in a firmly cross-linked RG-II–B–RG-II dimer, as seen in Figure5.

Furthermore, results from Mortimer *et al*. (2013) on *gonst1* (Golgi localised nucleotide sugar transporter) mutants altered in the mannosylation of mono- and dihexosylated GIPCs showed no decrease in RG-II dimerization rate. However, no difference in the Ara/GlcA ratio of the GIPCs of the mutants was observed, suggesting that GIPCs with more than three sugar residues were not affected by the mutation. Taken together with our evidence that the GIPCs with the strongest ability to bind B *in vivo* are highly glycosylated, it is likely that GIPCs potentially involved in RG-II dimerization are the (Pent)_*n*_-(Hex)_2_-HexA-Ins-P-Cer species.

### Co-expression analysis

**Figure 6:**
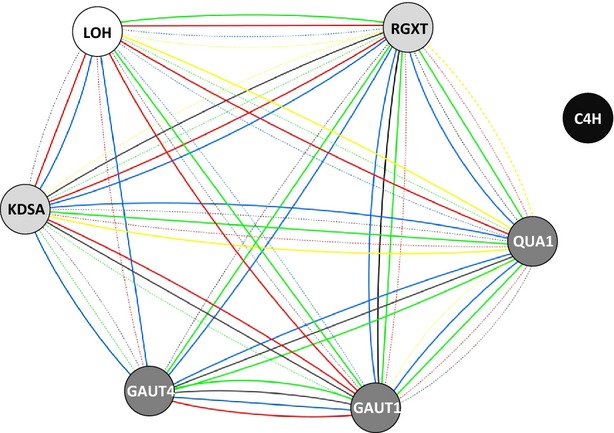
Co-expression network between genes involved in RG-II and glycosylinositol phosphorylceramide (GIPC) biosynthesis in various species using an edge-weighted force-directed approach, based on data retrieved from ATTED-II and visualised in Cytoscape 2.8 (http://www.cytoscape.org). Circles: white circle, LOH (encoding a ceramide synthase); pale grey, encoding enzymes involved in synthesis of RG-II side-chains; dark grey, encoding enzymes involved in synthesis of the homogalacturonan backbone; black, C4H (encoding cinnamate-4-hydroxylase, involved in lignin synthesis; included as a control). Lines: green lines, *Arabidopsis thaliana;* blue, *Populus trichocarpa;* red, *Oryza sativa;* black, *Glycine max;* yellow, *Zea mays*. Solid line, strong co-expression [mutual rank (MR) < 1000]; dotted line, weak co-expression (5000 > MR > 1000); no line, no transcriptomic data available or no co-expression (MR > 5000).

Results retrieved (Figure6) from Atted-II database (Obayashi *et al*., 2014) revealed that genes involved in GIPC biosynthesis, such as *LAG ONE HOMOLOGUE* (*LOH*) encoding a ceramide synthase (Ternes *et al*., 2011), were co-expressed in several species with genes involved in the biosynthesis of RG-II such as ketodeoxyoctulosonic acid [involved in biosynthesis of 3-deoxy-d-*manno*-2-octulosonic acid (KDO)] and *RGXT* (RG-II xylosyltransferase; involved in side-chain A biosynthesis). Likewise, *LOH* genes are co-expressed with genes involved in the biosynthesis of RG-II’s homogalacturonan backbone (*GAUT1, GAUT7* and *GAUT8,* encoding three galacturonosyltransferases). Moreover, both RG-II and GIPC biosynthesis occur in the Golgi apparatus (Mohnen, 2008); therefore, if the B-dependent dimerization of RG-II does require the intermediacy of a GIPC–B–RG-II complex, all necessary participants in the process would be present together in the endo-membrane/exocytosis system – which is the major location of RG-II dimerization *in vivo* (Chormova *et al*., 2013).

## Conclusion

Our results indicate that the essential element B, well known to contribute to cell-wall assembly by cross-linking pectic RG-II domains, also plays a structural role in plant lipid rafts. The plant-specific membrane components with which B interacts are proposed to be GIPCs on the evidence that their extractability is affected by *in-vivo* B supply, and by *in-vitro* treatment with cold dilute HCl (known to cleave borate diester bonds) or with borate buffer. We argue that the ability of high borate concentrations to disrupt lipid rafts may account for the phytotoxicity of excess soluble B which is encountered in certain soils especially in semi-arid areas. Furthermore, the electrophoretically observed *in-vitro* formation of a GIPC–RG-II complex, proposed to be established via a diol–B^(−)^–diol diester bridge, gives the first molecular explanation of the wall–membrane attachment sites that have been reported *in vivo*. Finally, our results suggest a synergistic role for GIPCs in the dimerization of pectic RG-II domains.

## Experimental Procedures

### *Rosa* cell-suspension cultures

Cell-suspension cultures of ‘Paul’s Scarlet’ rose [a complex hybrid; genus *Rosa*, initiated by Nickell and Tulecke (1959)], were grown as described by Chormova *et al*. (2013) in a medium that contained 3.3 μm H_3_BO_3_.

### Extraction of GIPCs

GIPCs were extracted according to a method adapted from Buré *et al*., 2011, yielding almost pure GIPCs. For this procedure, 1 g of rose cells was ground with a pestle and mortar in 4 ml ice-cold 0.1 m aqueous acetic acid. The slurry was filtered through acid-washed Miracloth (Calbiochem, http://www.merckmillipore.co.uk/life-science-research/calbiochem/), on a funnel and the residue washed with cold 0.1 m acetic acid. The filtrate was discarded and the residue was then incubated in 70% ethanol at 70°C for 15 min with or without 0.1 m HCl. The slurry was filtered hot through Miracloth and washed with 70% ethanol (±0.1 m HCl). The residue was re-extracted twice more in the same manner. The combined ethanolic filtrates were chilled immediately and left at −20°C overnight. The GIPC-containing precipitate was pelleted by centrifugation at 2000 ***g*** at 4°C for 15 min. The pellet was washed with ice-cold acetone until washes were non-acidic, and finally with cold diethyl ether, yielding a whitish precipitate, which was dried *in vacuo*. For studying the impact of B on GIPC extraction, the dried precipitate was directly phase-partitioned between butan-1-ol and water (±0.1 m HCl or 10 mm βMCD or 6 mm borate buffer pH 9.2 or ammonium buffer pH 9.2). For purifying GIPCs, the dried precipitate was suspended in 10 ml methanol/45% (aqueous) formic acid/chloroform (5:1:4 by volume) followed by 5 ml of water and homogenised. Whitish GIPC-containing material that collected at the aqueous/organic interface was dried and then phase-partitioned between butan-1-ol and aqueous 0.1 m HCl (1:1, v/v). Finally, the upper (butanol-rich) phase was dried and the residue was dissolved in 70% ethanol.

### ESI–MS and ESI–MS/MS

GIPC extracts were diluted 16-fold in 65:35 (v/v) isopropanol/water that contained 0.03% (w/v) ammonium acetate. Analyses were performed on a Q-TRAP mass spectrometer (Applied Biosystems, http://www.lifetechnologies.com) and samples infused at a flow rate of 7 μl min^−1^. ESI–MS/MS experiments were performed in accordance with Buré *et al*. (2011).

### Separation and purification of GIPCs by TLC

Lipids were chromatographed on Merck silica gel ‘60’ TLC plates in CHCl_3_:CH_3_OH:4 N NH_4_OH (9:7:2, v/v) with 0.2 m ammonium acetate_,_ a system described by Kaul and Lester (1975). In some cases the plate was immersed in 1% (w/v) di-sodium tetraborate decahydrate in MeOH for 15 sec and air-dried overnight before the samples were loaded. The chromatograms were sprayed with rhodamine 6G, which located the lipid spots, and with 1% sodium meta-periodate followed after a few minutes by 0.01% Schiff’s reagent (Sigma), which stains compounds with vicinal diol groups. After rhodamine spraying, glycolipid spots were specifically visualised by subsequent spraying with orcinol reagent (Skipski and Barclay, 1969). Relative band intensities were determined by use of ImageJ software (http://imagej.nih.gov/ij/).

### High-voltage paper electrophoresis

High-voltage paper electrophoresis (PE) of samples that contained [^3^H]RG-II was performed on Whatman No. 20 paper in pH 2 buffer [water/formic acid/acetic acid (45:1:4, by vol.)] (Fry, 2011) at 4.5 kV for 75 min or at 3.5 kV for 45 min. The papers were read with an AR-2000 radio-TLC Imaging Scanner (LabLogic, http://www.lablogic.com/).

### RG-II purification and radio-labelling

RG-II was prepared from suspension-cultured *Rosa* cells and monomerised as described (Chormova *et al*., 2013) and was kindly given by Dr Dimitra Chormova. In some experiments, we used monomeric [^3^H]RG-II of specific activity 17 MBq/μmol RG-II, also prepared by the method of Chormova *et al*. (2013).

### *In-vitro* RG-II dimerization and gel electrophoresis

*In-vitro* RG-II dimerization and gel electrophoresis were performed according to Chormova *et al*. (2013).
